# Supplementary material for: Diet and Depression during Peri- and Post-Menopause: A Scoping Review Protocol
Source: Methods Protoc. 2023 Oct 2;6(5):91. doi: 10.3390/mps6050091 (PMC10609501; doi:10.3390/mps6050091)
Supplement: Supplementary file 1 [file mps-06-00091-s001.zip › mps-2525709 Supplementary files/Supplementary file 2.pdf]

Supplementary file 2. Search strategy for all databases

Table S1. Full search strategy for Medline *(through Ovid)*

Theme or description		Search terms
Diet-related variables	1	*exp diet/* or *exp nutritive value/* or *exp hunger/*
	2	*nutritional physiological phenomena/* or *eating/* or *feeding behavior/* or *appetite regulation/*
	3	*food/* or *dairy products/* or *dietary carbohydrates/* or *dietary fats/* or *dietary fiber/* or *dietary proteins/* or *dietary supplements/* or *eggs/* or *fast foods/* or *fruit/* or *meals/* or *meat/* or *micronutrients/* or *nuts/* or *seeds/* or *vegetables/*
	4	diet*.ti,ab,kw.
	5	nutriti*.ti,ab,kw.
	6	food.ti,ab,kw.
	7	eat*.ti,ab,kw.
	8	energy intake.ti,ab,kw.
	9	(macronutrient* or micronutrient* or nutrient*).ti,ab,kw.
	**10**	**1 or 2 or 3 or 4 or 5 or 6 or 7 or 8 or 9**
Mental health	11	*exp depressive disorder/*
	12	*depression/* or *mood disorders/* or *anxiety/* or *stress, psychological/* or *mental health/* or *body image/*
	13	(depression* or (depressive adj3 (condition* or disorder* or symptom*))).ti,ab,kw.
	14	anxiety.ti,ab,kw.
	15	stress*.ti,ab,kw.
	16	mental health.ti,ab,kw.
	17	(body adj3 (image* or satisfact*)).ti,ab,kw.
	18	(risk* adj2 behavio?r*).ti,ab,kw.
	**19**	**11 or 12 or 13 or 14 or 15 16 or 17 or 18**
Peri-/post-menopausal women	20	*exp menopause/*
	21	menopaus*.ti,ab,kw.
	22	premenopaus*.ti,ab,kw.
	23	perimenopaus*.ti,ab,kw.
	24	postmenopaus*.ti,ab,kw.
	**25**	**20 or 21 or 22 or 23 or 24**
Combining search themes	**26**	**10 and 19 and 25**
Language	**27**	**limit 36 to (english or french)**
Search years	**28**	**limit 37 to yr="1993 -Current"**
Population specifications	**29**	**limit 38 to humans**
	**30**	**limit 39 to female**

Table S2. Full search strategy for EMBASE *(through Ovid)*

Theme or description		Search terms
Diet-related variables	1	*exp diet/* or *exp food intake/* or *exp feeding behavior/*
	2	*food/* or *dairy product/* or *dietary fiber/* or *dietary supplement/* or *egg/* or *fast food/* or *fat/* or *fruit/* or *health food/* or *meat/* or *nut/* or *sugar/* or *vegetable/*
	3	diet*.ti,ab,kw.
	4	nutriti*.ti,ab,kw.
	5	food.ti,ab,kw.
	6	eat*.ti,ab,kw.
	7	energy intake.ti,ab,kw.
	8	(macronutrient* or micronutrient* or nutrient*).ti,ab,kw.
	**9**	**1 or 2 or 3 or 4 or 5 or 6 or 7 or 8**
Mental health	10	*exp mental health/*
	11	*mood disorder/* or *depression/* or *anxiety/* or *mental stress/* or *body image/*
	12	(depression* or (depressive adj3 (condition* or disorder* or symptom*))).ti,ab,kw.
	13	anxiety.ti,ab,kw.
	14	stress*.ti,ab,kw.
	15	mental health.ti,ab,kw.
	16	(body adj3 (image* or satisfact*)).ti,ab,kw.
	17	(risk* adj2 behavio?r*).ti,ab,kw.
	**18**	**10 or 11 or 12 or 13 or 14 or 15 or 16 or 17 or 18**
Peri-/post-menopausal women	19	*exp "menopause and climacterium"/*
	20	*menopause/*
	21	menopaus*.ti,ab,kw.
	22	premenopaus*.ti,ab,kw.
	22	perimenopaus*.ti,ab,kw.
	24	postmenopaus*.ti,ab,kw.
	**25**	**19 or 20 or 21 or 22 or 23 or 24**
Combining search themes	**26**	**9 and 18 and 25**
Language	**27**	**limit 31 to (english or french)**
Publication years	**28**	**limit 32 to yr="1993 -Current"**
Population specifications	**29**	**limit 34 to humans**
	**30**	**limit 35 to female**

Table S3. Full search strategy for PsycINFO *(through Ovid)*

Theme or description		Search terms
Diet-related variables	1	*exp appetite/*
	2	*diets/* or *food intake/* or *eating behavior/*
	3	diet*.mp.
	4	nutriti*.mp.
	5	food.mp.
	6	eat*.mp.
	7	energy intake.mp.
	8	(macronutrient* or micronutrient* or nutrient*).mp.
	**9**	**1 or 2 or 3 or 4 or 5 or 6 or 7 or 8**
Mental healthc	10	*exp mental health/*
	11	*major depression/* or *dysthymic disorder/* or *recurrent depression/* or *treatment resistant depression/* or *anxiety/* or *psychological stress/* or *body image/*
	12	(depression* or (depressive adj3 (condition* or disorder* or symptom*))).ti,ab,mp.
	13	anxiety.ti,ab,mp.
	14	stress*.ti,ab,mp.
	15	mental health.ti,ab,mp.
	16	(body adj3 (image* or satisfact*)).ti,ab,mp.
	17	(risk* adj2 behavio?r*).ti,ab,mp.
	**18**	**12 or 13 or 14 or 15 or 16 or 17 or 18 or 19 or 20 or 21 or 22**
Peri-/post-menopausal women	19	Menopause/
	20	menopaus*.ti,ab,mp.
	21	premenopaus*.ti,ab,mp.
	22	perimenopaus*.ti,ab,mp.
	23	postmenopaus*.ti,ab,mp.
	**24**	**24 or 25 or 26 or 27 or 28**
Combining search themes	**25**	**11 and 23 and 28**
Language	**26**	**limit 21 to (english or french)**
Publication years	**27**	**limit 31 to yr="1993 -Current"**
Population specifications	**28**	**limit 32 to human**
	**29**	**limit 33 to female**

Table S4. Full search strategy for the Food Science and Technology Abstracts Database (*through Ovid*)

Theme or description		Search terms
Diet-related variables	1	*exp diet/* or *exp eating habits/* or *exp nutrients/*
	2	*appetite/* or *hunger/* or *satiety/*
	3	diet*.ti,ab,mp.
	4	nutri*.ti,ab,mp.
	5	food*.ti,ab,mp.
	6	eat*.ti,ab,mp.
	7	energy intake.ti,ab,mp.
	8	(macronutrient* or micronutrient* or nutrient*).mp.
	**9**	**1 or 2 or 3 or 4 or 5 or 6 or 7 or 8**
Mental health	10	*exp human psychology/*
	11	*mental health/*
	12	(depression* or (depressive adj3 (condition* or disorder* or symptom*))).ti,ab,mp.
	13	anxiety.ti,ab,mp.
	14	stress*.ti,ab,mp.
	15	mental health.ti,ab,mp.
	16	(body adj3 (image* or satisfact*)).ti,ab,mp.
	17	(risk* adj2 behavio?r*).ti,ab,mp.
	**18**	**10 or 11 or 12 or 13 or 14 or 15 or 16 or 17**
Peri-/post-menopausal women	19	*menopause/*
	20	menopaus*.ti,ab,mp.
	21	premenopaus*.ti,ab,mp.
	22	perimenopaus*.ti,ab,mp.
	23	postmenopaus*.ti,ab,mp.
	**24**	**21 or 22 or 23 or 24 or 25**
Combining search themes	**25**	**11 and 20 and 26**
Language	**26**	**limit 27 to (english or french)**
Publication years	**27**	**limit 28 to yr="1993 -Current"**
